# New Insights Into Interactions of Presynaptic Calcium Channel Subtypes and SNARE Proteins in Neurotransmitter Release

**DOI:** 10.3389/fnmol.2018.00213

**Published:** 2018-07-16

**Authors:** Rongfang He, Juan Zhang, Yiyan Yu, Laluo Jizi, Weizhong Wang, Miaoling Li

**Affiliations:** ^1^Key Laboratory of Medical Electrophysiology, Ministry of Education, Collaborative Innovation Center for Prevention and Treatment of Cardiovascular Disease, Institute of Cardiovascular Research, Southwest Medical University, Luzhou, China; ^2^Infectious Disease Department, The Affiliated Hospital of Southwest Medical University, Southwest Medical University, Luzhou, China; ^3^Department of Neurology, Liangshan Hospital of Integrated Traditional and Western Medicine, Xichang, China; ^4^Department of Physiology and Center of Polar Medical Research, Second Military Medical University, Shanghai, China

**Keywords:** Ca^2+^ channel subtypes, SNAREs, Ca^2+^ sensor, active zone, membrane fusion, neurotransmitter release

## Abstract

Action potential (AP) induces presynaptic membrane depolarization and subsequent opening of Ca^2+^ channels, and then triggers neurotransmitter release at the active zone of presynaptic terminal. Presynaptic Ca^2+^ channels and SNARE proteins (SNAREs) interactions form a large signal transfer complex, which are core components for exocytosis. Ca^2+^ channels serve to regulate the activity of Ca^2+^ channels through direct binding and indirect activation of active zone proteins and SNAREs. The activation of Ca^2+^ channels promotes synaptic vesicle recruitment, docking, priming, fusion and neurotransmission release. Intracellular calcium increase is a key step for the initiation of vesicle fusion. Various voltage-gated calcium channel (VGCC) subtypes exert different physiological functions. Until now, it has not been clear how different subtypes of calcium channels integrally regulate the release of neurotransmitters within 200 μs of the AP arriving at the active zone of synaptic terminal. In this mini review, we provide a brief overview of the structure and physiological function of Ca^2+^ channel subtypes, interactions of Ca^2+^ channels and SNAREs in neurotransmitter release, and dynamic fine-tune Ca^2+^ channel activities by G proteins (Gβγ), multiple protein kinases and Ca^2+^ sensor (CaS) proteins.

## Introduction

Influx of Ca^2+^ through presynaptic calcium channels into presynaptic terminals at active zone is a crucial step in synaptic vesicle exocytosis and rapid neurotransmitter release (Catterall, 2011). Interactions of Ca^2+^ channel and soluble N-ethyl-maleimide-sensitive factor attachment protein receptor (SNAREs) complex contribute to reduce the distance between vesicles and the presynaptic membrane (Catterall and Few, 2008). The close distance provides a spatial structure that can ensure triggering of the fast neurotransmitter release within milliseconds of the action potential (AP) arriving at the synaptic terminal (Südhof, 2013; Mochida, 2017). Changes in the kinetic properties of Ca^2+^ channels (such as channels open, close, inactivate and so on) directly or indirectly induce modulation of the exocytosis of the synaptic vesicle, and subsequently modulate the release of neurotransmitters in a negative or positive way (Atlas, 2013). Multiple subtypes of Ca^2+^ channels are present in the nervous system with diverse physiological functions (Mochida, 2018). Furthermore, a single neuron also contains different types of Ca^2+^ channel isoforms. Thus, the channel isoforms play a key role in integral regulation of the synaptic vesicle exocytosis. Until now, it has not been clear how the Ca^2+^ channel isoforms coordinate well and accurately regulate fast neurotransmitter release at synaptic terminals. In this mini review, we focus on the molecular structures and regulatory mechanisms of multiple Ca^2+^ channel isoforms, and the interactions of Ca^2+^ channels and SNAREs involving vesicles fusion and neurotransmitter releases.

## Diversity of Ca^2+^ Channels in Neurous System

The diverse subtypes of Ca^2+^ channels display different biological structures and distribution in the nervous system. The diversity of channels corroborates its different physiological functions.

**Figure 1:**
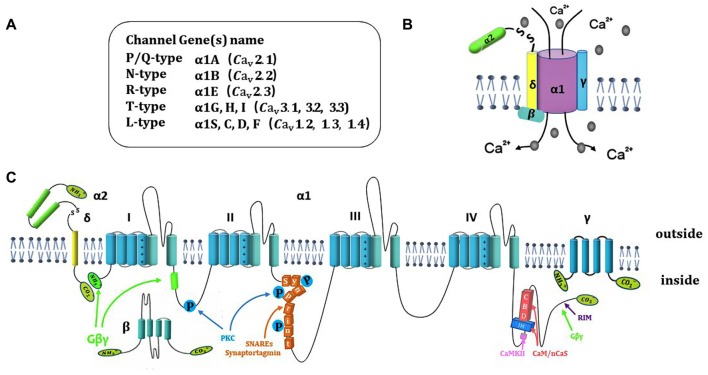
Ca^2+^ channel structure, organization and interaction with regulatory proteins. **(A)** Summary of 10 different subtypes of Ca^2+^ channel. **(B)** Representation of subunits composition of Ca^2+^ channels and auxiliary subunits. **(C)** The subunit consists of four homologous domains (I–IV), auxiliary subunits and interaction of Ca^2+^ channel subunits with regulatory proteins include SNARE proteins, Gβγ, kinase and CaS proteins.

According to the unique electrophysiological and pharmacological properties, voltage-gated calcium channel (VGCC) have been classified into N-, P/Q-, R-, L- and T-type (Ertel et al., 2000). N-, P/Q-, R- and L-type is termed as high-voltage activated Ca^2+^ channel, while T-type is low-voltage activated Ca^2+^ channel (<−40 mV). High-voltage activated Ca^2+^ channels are composed of the pore-forming Ca_v_α1 and four auxiliary subunits (Ca_v_α2, Ca_v_β, Ca_v_γ and Ca_v_δ; Catterall, 2000), while T-type contains only the Ca_v_α1 subunit (Ca_v_α1G, Ca_v_α1H and Ca_v_α1I; Figures 1A,B). The neuronal Ca_v_α1 subunit (190–250 kDa) is the largest and main subunit, which is composed of about 2000 amino-acid residues. The molecular weights of α2, β, γ and δ subunits are 143 kDa, 53–70 kDa, 30 kDa and 24–27 kDa, respectively. The Ca_v_α1 contains four homologous domains (I–IV; Figure 1C), and each domain of Ca_v_α1 is comprised of six transmembrane α helices (S1–S6). The transmembrane S5–S6 segments form a p loop, and the S1–S4 segments serve as the voltage sensor (Yu et al., 2005). Diversity of Ca_v_α1 isoforms determine the channel subtypes. Ten different types of Ca^2+^ channels have been identified (Yu and Catterall, 2004). The Ca_v_α1 subunit genes are classified as Ca_v_1.1–1.4 (L-type), Ca_v_2.1–2.3 (P/Q-, N-, and R-type) and Ca_v_3.1–3.3 (T-type; Figure 1B), each of them belongs to CACNA1x gene families. N-type and P/Q-type Ca^2+^ channels are the main Ca^2+^ channels in nerve terminals and play an important role in fine-tuning of rapid neurotransmitter release at synaptic terminals (Ariel et al., 2012). The R-type (Ca_v_2.3) Ca^2+^ channels are present in the peripheral nervous system (PNS) and central nervous system (CNS). Though R-type Ca^2+^ channels are not the main Ca^2+^ channels, they are also involved in presynaptic plasticity and neurotransmitter release (Breustedt et al., 2003; Dietrich et al., 2003; Naidoo et al., 2010). T-type Ca^2+^ channel present in peripheral, central synapses and neuroendocrine cells, play a key role on tuning of basal neurosecretion near resting potential with a mild stimulation (Lambert et al., 2014).

The auxiliary subunits of Ca^2+^ channels include α2, β, γ and δ subunits (Figure 1A). The α2 and δ subunits are encoded by the same gene that bind together with disulfide linkage to form α2-δ subunit complex. α2-δ subunit exerts a role of increased calcium current and upregulation of gene expression. The interactions of α2-δ subunit with extracellular Ca_v_α1 modulate the binding of divalent cations (Cantí et al., 2005). α2δ-1 isoform is encoded by the gene Cacna2d1. The interaction of α2δ-1 and NMDA receptors significantly increased in neuropathic pain (Dolphin, 2012; Patel et al., 2013). Chen et al. (2018) demonstrate that gabapentin reduces neuropathic pain by inhibiting of the interaction between the C terminus of α2δ-1 and NMDA receptors. The whole β subunit is located in cytoplasm. The functional role of β subunit is to ensure the α1 subunit binding to the plasma membrane and prevent it trafficking to the endoplasmic reticulum. β subunit regulates membrane protein expression and gating of Ca^2+^ channels (Arikkath and Campbell, 2003). The γ subunit comprises of four transmembrane α helices, that can slightly reduce Ca^2+^ current density and change kinetic properties by interacting with the Ca_v_α1 subunit (Osten and Stern-Bach, 2006). The specific polypeptide toxins from snail and spider venoms, block multiple Ca^2+^ channel subtypes. ω-conotoxin GVIA (ω-Cgtx GVIA) blocks the N-type channels irreversibly in central nervous system (CNS) and Peripheral nervous system (PNS; Prashanth et al., 2014). ω-agatoxin IVA (ω-AgaIVA) blocks the Ca_v_2.1 (P-type) and ω-Aga IVA blocks the Ca_v_2.1 (Q-type) with a lower affinity in CNS (Arranz-Tagarro et al., 2014; Ricoy and Frerking, 2014). ω-conotox in MVIIC is a toxin from the venom of marine conus snail, which targets Ca_v_2.1 with high affinity and targets Ca_v_2.2 with low affinity (Catterall et al., 2005). α-conotox in Vc1.1 does not affect Ca_v_2.1 but strongly inhibits Ca_v_2.3 Ca^2+^ channels through GABA_B_ receptor (Berecki et al., 2014). Ca_v_2.3 Ca^2+^ channels were potently blocked by Zn^2+^ (IC_50_ = 0.78 ± 0.07 μmol/L; Traboulsie et al., 2007). The tetraline derivative of mibefradil and the peptide blocker of scorpion toxin kurtoxin have been evaluated as potential Ca_v_3 Ca^2+^ channel inhibitors in CNS and PNS (Chuang et al., 1998).

The diversity of Ca_v_α1 and auxiliary subunits confirms distinct molecular structures, synaptic properties and distributions that are involved in the regulation of various physiology functions in neurotransmitter release. P-/Q-type Ca^2+^ channels mediated GABA release in the most of GABA releasing inhibitory neurons (Lonchamp et al., 2009). Glutamate-release is often mediated by integrated interactions of P-/Q- and N-type Ca^2+^ channels in the vast majority of glutamatergic cortical and cerebellar synapses (Ladera et al., 2009). Furthermore, P-/Q-type Ca^2+^ channels decrease fusion pore stability and trigger vesicle fusion, N-type and L-type Ca^2+^ channels slow down fusion pore expansion (Ardiles et al., 2007). In the axon terminal, P-/Q- type Ca^2+^ channels are close to the release zone than other Ca^2+^ channels in various synapses. As a result, P-/Q- type Ca^2+^ channels (Cav2.1) may lead to higher local presynaptic Ca^2+^ concentrations and frequently co-localized with synaptotagmin-containing vesicle clusters, whereas the N-type channel (Cav2.2) and R-type channel (Cav2.3) are only partially involved in vesicle clusters (Wu et al., 1999). The significant role of N-type Ca^2+^ channels is involved in neurotransmitter release in cortical and hippocampal synapses. L- and T-type Ca^2+^ channels are involved in neurotransmission release in various retinal neurons. T-type Ca^2+^ channels play a crucial role in neurotransmitter release and its regulation in special reciprocal synapses. Functionally, P-/Q-type Ca^2+^ channels may be mainly related to fast, synchronous exocytosis, and N-type Ca^2+^ channels may contribute to exocytosis in neurons processing information, P-/Q-type Ca^2+^ channels have been shown to be more efficient in neurotransmitter release than N-type Ca^2+^ channels in most investigated synapses, as in entorhinal stellate neurons, different inhibitory interneurons, cerebrocortical synapses or cerebellar parallel fiber terminals (Ladera et al., 2009).

## Interactions of Ca^2+^ Mediated Membrane Fusion by SNARE Proteins and Active Zone Proteins

**Figure 2:**
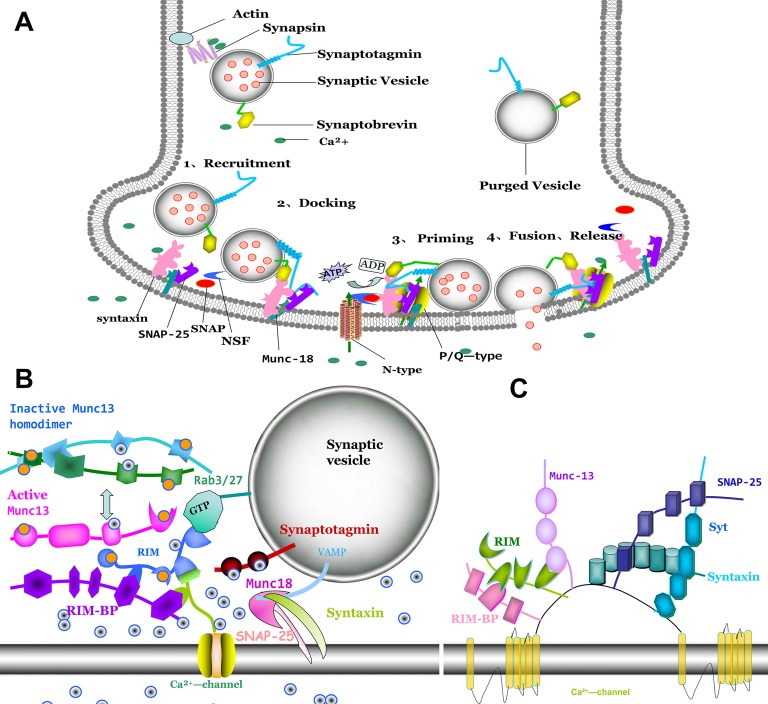
Molecular model of synaptic vesicle fusion machinery, interactions of active zone proteins, presynaptic Ca^2+^ channel and SNAREs. **(A)** The process of vesicle fusion: 1. synaptic vesicle recruiting to active zone; 2. synaptic vesicle docking at the presynaptic membrane and with SNAREs complex conformation. 3. priming of synaptic vesicle on presynaptic membrane; and 4. fusion pore to open and with neurotransmitter release. **(B)** Direct interaction of Cav2 α1 subunits with proteins Rab3 interacting molecules (RIM), RIM-binding protein (RIM-BP), Munc-13 and SNAREs (syntaxin, SNAP-25 and synaptobrevin). **(C)** RIM binding to RIM-BP induced Munc13 from inactive homodimer to active heterodimer, which promoted Sec1/Munc18-1 (SM) protein dissociated with syntaxin-1. Syntaxin-1 changes from closed formation to open formation. Syntaxin-1 and SNAP-25 interacted with synaptobrevin to form SNAREs. Ca^2+^ entry through Ca^2+^ channel induced interaction with synaptotagmin, which trigger vesicle fusion.

Ca^2+^ entry through presynaptic Ca^2+^ channels can trigger vesicle fusion by assembly of the SNARE proteins complex [t-SNARE proteins syntaxin-1 and SNAP-25, v-SNARE protein synaptobrevin (VAMP)] (Südhof, 2004; Bao et al., 2018; Figure 2A). SNARE function is widely reported to be associated with the processing of physiology and pathophysiology. It is reported that modifying SNARE function through regulating exocytosis can provoke metabolic diseases such as obesity (Valladolid-Acebes et al., 2015), which is improved by many therapies such as exercise training (Ramos-Miguel et al., 2015; Roh and So, 2017; Roh et al., 2017). The release of neurotransmitter requires localization of both calcium channels and synaptic vesicle proteins to the presynaptic active zone (Südhof, 2012). Rab3 interacting molecules (RIM) localizes in active zone (Figure 2B), which contain an N-terminal zinc finger domain, a central PDZ domain, C-terminal C2A and C2B domain and a conserved sequence between the two C-terminal domains (Wang and Südhof, 2003). RIM plays an essential role for synaptic vesicle docking and priming (Deng et al., 2011; Han et al., 2011). Munc13-1 is a large multidomain protein in active zone that plays a central role in synaptic vesicle priming (Brose et al., 1995; Augustin et al., 1999; Fukuda, 2003). The interaction of SNARE and SM (sec1/Munc18) proteins control the millisecond timescale presynaptic fusion after AP. Before priming, the Munc13 C2A-domain forms a constitutive homodimer (inactive state; Figure 2C). When Munc13 transforms from inactive state to an active state, Munc13-1 switches from a homodimer to a heterodimer (Munc13-1-RIM), may regulate synaptic vesicle priming (Lu et al., 2006). RIM-binding proteins (RIM-BPs) are also large multidomain proteins (~200 kDa) in active zone, that tightly bind to RIM. PDZ-domain of both RIM and RIM-BPs bind to Ca^2+^-channels for tethering Ca^2+^ channels to an active zone (Han et al., 2011; Kaeser et al., 2011). Deletion of RIM or RIM-BP (Liu et al., 2011; Kaeser et al., 2012) causes loss of Ca^2+^ channels from active zone and decreases Ca^2+^ entry. The central PDZ-domain of RIM can bind directly with N-type and P/Q-type, without binding with L-type Ca^2+^ channels (Kaeser et al., 2011). RIM that lacks the PDZ-domain exhibits loss binding abilities with Ca_v_ channels. Ca_v_ channels are recruited to active zone for synaptic vesicle fusion by a tripartite complex formation (RIM, RIM-BP and the C-terminal tail of Ca^2+^ channels) that needs assistance by Munc13-1. Munc13-1-RIM heterodimer formation is a key component for fusion. Furthermore, the C2B domain of RIM can modulate Ca^2+^ channel activation (Kaeser et al., 2012). Recently, it was reported that Munc13, independent with Munc18, promotes the syntaxin-1-synaptobrevin complex formation during the assembly of the triplet SNARE complex. Interaction with Munc18 and Munc13 contributes to syntaxin/SNAP-25 complex formation (Lai et al., 2017).

Before forming the SNARE complex (Figure 2A), syntaxin-1 is presented in a closed conformation by interaction with SM proteins which cannot promote SNARE complex formation. When the zinc-finger of RIM binds to the C2A domain of Munc13, Munc13 is activated by homodimer dissociation. Subsequently, the activation of Munc13 drags RIM closer to the presynaptic membrane. Ma et al. (2011) have demonstrated that Munc13 can accelerate the transfer from the closed syntaxin-1-Munc18-1 heterodimer to an open syntaxin-1 for promoting SNARE complex formation. SM proteins are fundamental for synaptic vesicle trafficking. However, another study reported that the SM proteins exert no effect on spontaneous fusion and Ca^2+^-triggered fusion with SNAREs, complexin-1 and syt-1 (Zhang et al., 2015). Stable SNAREs complex provide energy for membrane fusion (Weber et al., 1998).

Complexin is a small soluble protein that controls (activates or suppresses) the trigger-release and spontaneous release (Fernández-Chacón et al., 2001; Pang et al., 2006; Mohrmann et al., 2015; Yu et al., 2018). The central helix of complexin binds to the interface of the v- and t-SNAREs close to the membrane (Fernández-Chacón et al., 2001; Chen et al., 2002; Tang et al., 2006). Complexin displays an activated effect in fast synchronous release and an inhibited effect in spontaneous release (Maximov et al., 2009; Kaeser-Woo et al., 2012). The synchronous function of complexin-1 is promoted by interactions with the SNARE complex at the N-terminal, whereas the suppressive action of spontaneous fusion is involved in binding with the C-terminal domain of complexin-1, but not the N-terminal domain (Lai et al., 2014). Lai et al. (2016) have demonstrated the mechanism that the N-terminal domain of the complexin can independently modulate the interaction of presynaptic membrane and the SNAREs. Furthermore, Gong et al. (2016) have revealed that the C-terminal domain is pivotal for regulation of spontaneous release and suppression of Ca^2+^-independent fusion in a curvature-dependent phase. Misplacement of complexin to the plasma membrane increases the variableness and the mean decay time constant of synchronization with NMDA-type glutamate receptor initiated postsynaptic currents.

Synaptotagmin (syt) is a Ca^2+^ sensor that can evoke fast and synchronous neurotransmitter release (Xu et al., 2007). Syt-1 contains two homologous Ca^2+^ sensor modules: C2 domains (C2A and C2B) and transmembrane domain. Syt-1, Syt-2 and Syt-9 bind to Ca^2+^ to promote synchronous transmitter release, while Syt-7 evokes a asynchronous transmitter release (Bacaj et al., 2013; Brewer et al., 2015; Zhou et al., 2015; Pérez-Lara et al., 2016).

Ca^2+^ binding to syt abolishes the complexin clamp and triggers synaptic vesicle fusion. Recent study has revealed that complexin may regulate fusion in cooperation with Syt. Syt1-SNARE and complexin-SNARE cooperate to activate synchronous release and regulate synchronous release after the AP has arrived at the synaptic terminal (Jorquera et al., 2012; Dhara et al., 2014). Recent study has demonstrated that the tripartite SNARE complexin-syt-1 complex at a synaptic vesicle docking site exerts an open state for trigger fusion. Interaction of interfaces are fundamental for Ca^2+^-triggered neurotransmitter release. Disruption of tripartite interface cannot trigger neurotransmitter release, although the primary interface is intact. It implied that both the primary and tripartite interfaces are required for Ca^2+^-triggered synaptic vesicle fusion (Akyuz et al., 2013; Gipson et al., 2017). Before the Ca^2+^ trigger, syt interacts with SNARE proteins in the targeted membrane to prevent SNARE complex assembly (Chicka et al., 2008). Ca^2+^ entry through Ca^2+^ channels increases the affinity of syt-1 with syntaxin-1 for approximately two orders of magnitude (Chapman et al., 1995; Bhalla et al., 2006). Munc13, notably, enhances the transforms from the Munc18-1 syntaxin-1 complex to the SNARE complex (Ma et al., 2011) that can open the closed form of the SNARE protein (Lu et al., 2006; Kaeser et al., 2011). NSF (Sec18) and α-SNAP (Sec17) form a molecular chaperone for dynamic modulation of the disassembly of cis-SNAREs. Recently, Song et al. (2017) demonstrated that Sec17 residue K159 contributes to enhance the synaptic vesicle fusion. Furthermore, Sec18 can augment the interactions of Sec17 and trans-SNARE (Schwartz et al., 2017; Song et al., 2017). Lai et al. (2013) also have found that syt1 and Ca^2+^ are required for pore formation and expansion. Furthermore, SNAREs alone are sufficient in promoting membrane hemifusion.

## Interaction of Ca^2+^ Channel Subtypes and SNARE Proteins Complex

Presynaptic VGCCs have been classified into three super families (Ca_v_1, Ca_v_2 and Ca_v_3). Ca_v_2 (P/Q-, N- and R-type) are the dominant channel subtypes for fast presynaptic transmitter. Ca_v_2.2 (N-type) interacts with active zone proteins (RIM, RIM-BP) and SNAREs to regulate the vesicle fusion. RIM-C2A and RIM-C2B bind the pore-forming subunit of N-type Ca_v_ channels in a Ca^2+^-independent manner that weakly interacts with the Ca_v_1.2 (L-type, α1c), but do not interact with the Ca_v_1.3 (L-type, α1D). Furthermore, RIM (C2 domain) enhances the interaction with syt-1 when intracellular Ca^2+^ concentration is increased. Removal of RIM domain heavily reduces the channel current and number of docking vesicles resulting in decreasing Ca^2+^ channel coupling with vesicle. The central PDZ-domain of RIM interacts with the C-terminal of presynaptic N- and P/Q-type Ca^2+^-channels, with no act on L-type Ca^2+^ channels. Deletion of RIM inhibits most neurotransmitter release due to impairing the synaptic priming and decreasing the Ca^2+^ channels localization in presynaptic membrane (Kaeser et al., 2011; Han et al., 2015). It is well-known that vesicle priming and Ca^2+^ influx do not require RIM C2B domains. Recently, studies have found that C2 domains of RIM do not bind to Ca^2+^, but bind to PIP2. PIP2 binding to RIM C2B domains exerts a critical role for vesicle priming and Ca^2+^ channel tethering to PIP2 containing targeted membranes (de Jong et al., 2018).

Active zone scaffold protein Bassoon directly binds to RIM-BP to modulate synaptic vesicle docking via an indirect contact with Ca_v_2.1. Genetic deletion of Bassoon or an acute interference with Bassoon RIM-BP interaction reduces synaptic amount of Ca_V_2.1, which gently regulates P/Q-type Ca^2+^ current to trigger synaptic transmission (Davydova et al., 2014). Both genetic ablation of Bassoon or interference of the link between Bassoon and RIM-BP reduced the numbers of Ca_v_2.1 in active zone, decelerated AP-triggered neurotransmitter release and impaired the synaptic transmission. Ca_v_2.2 current was increased for compensation for Ca_v_2.1-induced decreases (Acuna et al., 2015). RIMs-mediated vesicle priming is not produced by coupling with Munc13, whereas it is directly activated by Munc13. Zn^2+^ finger domain of RIM binds to Munc13 to promote vesicle priming, thereby dissociating Munc13 from heterodimer to homodimer and promotes priming in Munc13-deficient synapses. Hence, homodimer of Munc13 inhibits priming, and RIM activates priming by disrupting Munc13 homodimer (Deng et al., 2011). At rod photoreceptor ribbon synapses, RIM causes a dramatic loss of Ca^2+^ entry through Ca_v_1.4 channels and reduces trigger release. RIM induces Ca^2+^ entry, which in turn promotes release by modulating Ca_v_1.4 channel opening (Grabner et al., 2015). Alternative splicing (exons of 44 and 47) of Ca_v_2.1α1 (P/Q-type) induces gene variants of the C-terminal region (CTD) of Ca_v_2.1. The two exons interact with RIM (1α and 2α), impair the binding of CTD with RIM and implied suppressive effect of RIM on voltage-dependent inactivation (Hirano et al., 2017). Syntaxin, SNAP-25 and syt-1 possess specific “synprint” binding site interaction with Ca_V_2.1 and Ca_V_2.2 at the intracellular loop linking domains II and III (LII-III; Figure 1C). Diversity of VGCC types display distinct tissue specificity, subcellular localizations, kinetics performance and amount of Ca^2+^ influx. Ca_V_2.1 is the most abundant expression in neurons (Cabañes, 2008; Catterall and Few, 2008; Davies et al., 2011; Jahn and Fasshauer, 2012; Davydova et al., 2014; Wang and Augustine, 2014; Chai et al., 2017; Silva et al., 2017). GSK-3β displays inhibitory effects in presynaptic vesicle exocytosis by phosphorylating Ca_V_2.1 and disturbing SNARE complex formation. A mutation in the first intracellular loop of Ca_V_2.1 prevents interaction with SNARE proteins and impair SNAREs complex formation. SNAREs proper interact with synprint site to help vesicles docking near the Ca^2+^ entry pathway, and modulate steady-state inactivation of Ca_v_2.1 (Serra et al., 2018). R-type (Ca_v_2.3) channels are localized at the presynaptic terminal and trigger neurotransmitter release by enhancing presynaptic Ca^2+^ levels. Wu et al. (1999) reported that R-type (Ca_v_2.3) Ca^2+^ channels contributed to about 26% of the total Ca^2+^ current during a medial nucleus of the trapezoid body presynaptic AP, but display a lower efficacy than other types of Ca^2+^ channels. R-type Ca^2+^ channels are also involved in fast synaptic excitation (Naidoo et al., 2010). Recently, researchers revealed that R-type Ca^2+^ channels linked with NOS to induce NO release by controlling gastrointestinal smooth muscle relaxation in the guinea pig ileum via a purine transmitter (Rodriguez-Tapia et al., 2017).

Aplysia pleural sensory neurons are involved in the forms of presynaptic plasticity. The Aplysia Ca_V_2α1 subunit EF-hand tyrosine Y1501 are targets for modulation by GPCRs through Src kinase. The heterosynaptic depression of the Ca_V_2 channel current is inhibited when channel is combined with a Y-F mutation at the conserved Src phosphorylation. It implies that the inhibition of the Ca_v_2 calcium current is partially, at least, responsible for the inhibition of neurotransmitter release with heterosynaptic depression (Dunn et al., 2018). Ca^2+^ channels are also involved in nerve injury. Lu et al. (2018) first demonstrated that lycopene depress glutamate release through inhibition of voltage-dependent Ca^2+^ entry (N-type and P/Q-type channels) and protein kinase C in rat cerebrocortical nerve terminals and not by intracellular Ca^2+^ release.

The Ca_V_3 family Ca_V_3.1(α1G), Ca_V_3.2(α1H), and Ca_V_3.3(α1I) mediate T-type Ca^2+^ currents. T-type channels have been revealed to regulate neurotransmitter release in central, peripheral synapses and neuroendocrine cells that modulate basal neurosecretion close to resting potential with mild stimulations. Although T-type channels have no directly binding peptide (no synprint binding site), Ca_v_3.2 channels interact with syntaxin 1A and SNAP-25. The interactions form nanodomains that can be regulated transiently and low voltages controlling neural activity and neuroendocrine. Interaction of T-type channels, secretory vesicles, and SNAREs form a nanodomains complex. T-type Ca^2+^ channels can directly interact with SNAREs (syntaxin 1A-Ca_v_3.2-SNAP25) to control exocytosis. It is clear that T-type channels contribute to synaptic transmission in neurons and neuroendocrine cells under conditions of rest and mild stimulation. T-type Ca^2+^ channels are also involved in the development of a neuropathic pain. T-type Ca^2+^ channel subunit Ca_V_α2δ interaction with the extracellular matrix protein thrombospondin-4 (TSP4) contributes to initiate, but not for the maintenance of excitatory synaptogenesis. Treatment with gabapentin blocks the early pain state but does not reverse the delayed state. It implies that early intervention with gabapentin may prevent the development of injury-induced chronic pain, one of the reasons is that Ca_V_α2δ1/TSP4 initiates abnormal synapse formation (Yu et al., 2018).

Interestingly, Diao et al. (2013) have found that native presynaptic protein α-Synuclein (α-Syn) has little effect on Ca^2+^-triggered synaptic fusion efficiency or kinetics in neurotransmitter releases. On the contrary, α-Syn plays a key role in clustering of v-vesicles. Parkinson’s disease induces α-Syn mutant at A30P. Pathogenic α-Syn reduces the clustering ability that resulted in affecting neurotransmission (Diao et al., 2013). Furthermore, N-terminal acetylation can significantly decrease α-Syn oligomerization that can preserve its native conformation against pathological aggregation (Bu et al., 2017).

## Ca^2+^ Channels Regulation and Synaptic Transmission

The activity of presynaptic calcium channels is also modulated by βγ-subunits of G proteins (Gβγ), protein kinases (PKC, CaMKII) and Ca^2+^ sensor (CaS) proteins. Gβγ negatively regulates the neurotransmitter release by inhibition of Ca_V_2 (P/Q- and N-type) Ca^2+^ channels in synaptic terminals. Gβγ directly binds to Ca_V_2.2α1 at the N-terminal_45–55_ (Cantí et al., 1999), the intracellular loop domains between I and II (LI-II) at 377393 (Zamponi et al., 1997) and the C-terminus at 22572336 (Li et al., 2004). Only the N-terminal can suppress Ca_V_2 channels activity. The site at the N terminus and intracellular loop (LI-II) produces a more potent effect (Stephens and Mochida, 2005; Figure 1C). Furthermore, it has also been demonstrated that the Ca_V_2.2 alternative splicing isoform, e37a, exerts an increase in the expression of N-type Ca^2+^ channels and also increases the channel opening compared to Ca_v_2.2 channels that contain e37b (Castiglioni et al., 2006). Injection of N-terminal or a I-II loop interaction domain peptide into sympathetic superior cervical ganglion (SCG) neurons attenuates noradrenaline-initiated G protein regulation, and reduces synaptic transmission, and decreases Ca^2+^ current density. Furthermore, mutation at N-terminal abolishes the inhibitory effects of the N-terminal peptide (Bucci et al., 2011). Gβγ binding to N-terminal and loop I–II of Ca_V_2.2 contributes to regulate the function of Ca_V_2.2. Interestingly, the SNARE protein syntaxin 1A co-localizes with Ca^2+^ channels and Gβγ. Co-expression of syntaxin 1A with N-type channels induces tonic inhibition mediated by Gβγ (Jarvis et al., 2000). Nevertheless, syntaxin 1B does not display such effect (Lü et al., 2001). It is suggested that the spatial localization of the G protein-synaprint-Ca_V_2.2 complex is critical for neurotransmitter release (Yoon et al., 2008). The synaptic protein cysteine string protein promotes interaction between G proteins and the synprint site on Ca_V_2.2 channel for enhancing neurotransmitter release (Figure 2C).

Protein kinases (such as PKC and CaMKII) are localized in presynaptic terminals that can phosphorylate both Ca^2+^ channels and SNAREs (Figure 2C). PKC and CaMKII-phosphorylation of Ca^2+^ channels at the synprint site induce forceful inhibition of its binding to syntaxin-1A and SNAP-25 (Yokoyama et al., 1997). Phosphorylation of Ca^2+^ channels at the synprint by PKC is located at serines 774 and 898 which resulted in modulating the interaction with syntaxin-1A and SNAP-25. However, PKC phosphorylation failed to dissociate Ca_V_2.2/syntaxin 1A complexes. Auxiliary subunits of Ca_v_2.2 also participate in regulation of the function of Ca_v_2.2 channel and then modulate the transmitter release. The acetyl-β-methylcholine (MCh) or PKC isozymes (βII or ε) are unable to potentiate Ca_v_2.2 current in the presence of Ca_V_β subunits. Cavβ subunits complete suppression of the interactions between PKC and Ser/Thr sites of Ca_v_2.2α1 subunits (Thr-422, Ser-425, Ser-1757, Ser-2108 and ser-2132; Rajagopal et al., 2014). The mutation of PKC sites (Thr-422, Ser-1757 and Ser2132) can abolish MCh potentiation on Cav2.2α1 currents. The stimulatory sites at Thr-422, Ser-2108 or Ser-2132 and inhibitory sites at Ser-425 of Cav2.2α1 are identified by binding to PKCs βII and ε subunits. Whereas, the stimulatory sites at Thr-365, Ser-1995 and Ser-2011 and the inhibitory sites at Ser-369of Ca_v_2.3α1 subunits are homologous with Cav2.2α1. The stimulatory effects of PKC at the site of Thr-365 or Ser-1995 were fully offset by inhibitory site at Ser-369. PKC cannot inhibit the effects via the coexistence with Thr-365 and Ser-1995 (Rajagopal et al., 2017).

The phosphorylation of Core-conserved residues inside the SNARE domain can suppress vesicle fusion. Studies revealed that secretory protein VAMP8 phosphorylation by PKC at multiple residues in the SNARE domain mediated vesicle fusion, where protein kinase activation decreases and phosphatase activation increases the capacity of VAMP8 (Malmersjö et al., 2016).

CaMKII potently inhibits the interactions between syntaxin-1A and SNAP25 by phosphorylation at Ser 784 and 896 (Yokoyama et al., 2005). Each site of phosphorylation modulates syntaxin-1 and SNAP-25 binding to the synprint site. PKC or CaMKII phosphorylates Ca_v_α1 at the synprint sites that manipulates a biochemical switch for controlling the interaction of synprint and SNAREs. It implied that switch role provides a potential functional link between neurotransmitter release and protein phosphorylation for tethering and docking synaptic vesicle in an optimal position to respond to the Ca^2+^ signal from presynaptic Ca^2+^ channels (Catterall and Few, 2008).

In neurons, multiple Ca^2+^ sensor (CaS) proteins are involved in neuronal Ca^2+^ signaling transmitter. The distance between voltage-gate Ca^2+^ channels and CaS for exocytosis determines the timing and probability of neurotransmitter release (Nakamura et al., 2018). Calmodulin (CaM) is one of the members of a subfamilies of CaS proteins. Vesicle protein synaptotagmin is also a CaS protein for fast neurotransmission. Interactions of Ca^2+^/CaM binding to the CaM-binding domain (CBD) and IQ-like motif (IM) of Ca_V_2.1 contribute to facilitate and inactivate Ca_v_2.1 channels. Mutation of the motifs of CBD and IM prevents synaptic facilitation. Nanou et al. (2018) demonstrate a direct link between regulation of Ca_V_2.1 channels and short-term synaptic plasticity in native hippocampal excitatory and inhibitory synapses. CaBP1 and VILIP-2 are neurospecific CaM-like CaS proteins that potently modulate Ca_V_2.1 channels function. Ca^2+^-binding protein (CaBP1), Visinin-like protein 2 (VILIP-2) and neuronal calcium sensor-1 (NCS-1) are the key CaS proteins for synaptic transmission. CaBP1 is highly expressed in the brain and retina, and co-localized in the CBD of Cav2.1α1 (Lee et al., 2002). CaBP1 binds to CBD in a Ca^2+^ independent profile. Leal et al. (2012) demonstrated that CaBP1 performed a blockade effect on Ca^2+^-dependent facilitation of Cav2.1, and reduced facilitation of synaptic transmission in superior cervical ganglion neurons. Nanou et al. (2018) also demonstrated CaBP1/caldendrin as the CaS protein interacting with Ca_V_2.1 channels to mediate rapid synaptic depression in the inhibitory hippocampal synapses. On the contrary, VILIP-2 blocked Ca^2+^-dependent inactivation of Ca_V_2.1 current, and notably reduced synaptic depression and showed increasing facilitation. VILIP-2 is highly expressed in neocortex and hippocampus, and plays a complementary effect on CaBP1. These studies reveal that CaBP1 and VILIP-2 bind to the same site with opposite effects on Cav2.1. The integrated effect contributes to modulating short-term synaptic plasticity (Leal et al., 2012; Catterall et al., 2013). The N-terminal myristoylation site and EF-hand motifs of CaBP1 and VILIP-2 determine their differential regulated role on Ca_V_2.1 channels. CaS proteins serve as bidirectional switch that fine-tune the relationships of Ca_V_ and synaptic transmission. Thereby, the balance between facilitation and depression is a key role on neurotransmitter release (Leal et al., 2012).

Neuronal calcium sensor-1 (NCS-1) has been also shown to enhance synaptic facilitation. NCS-1 directly interacts with IQ-like motif and CBD site at the C-terminal domain of Ca_V_2.1. NCS-1 reduces Ca^2+^-dependent inactivation of Ca_v_2.1 through interaction with the IQ-like motif and CBD. NCS-1 modulates Ca^2+^ current amplitude or kinetics activity. These studies indicate that NCS-1 directly binds to Ca_V_2.1 to serve short-term synaptic facilitation and confirm that CaS proteins are crucial in fine-tuning short-term synaptic plasticity (Yan et al., 2014).

## Author Contributions

RH, JZ, YY and LZ contributed to the review of the literature, and editing of the manuscript. WW and ML wrote the draft manuscript. All authors read and approved the submission.

## Conflict of Interest Statement

The authors declare that the research was conducted in the absence of any commercial or financial relationships that could be construed as a potential conflict of interest.
